# RAPSearch: a fast protein similarity search tool for short reads

**DOI:** 10.1186/1471-2105-12-159

**Published:** 2011-05-15

**Authors:** Yuzhen Ye, Jeong-Hyeon Choi, Haixu Tang

**Affiliations:** 1 School of Informatics and Computing, Indiana University, Bloomington, IN 47408, USA; 2 Center for Genomics and Bioinformatics, Indiana University, Bloomington, IN 47405, USA

**Keywords:** short reads, similarity search, suffix array, reduced amino acid alphabet, metagenomics

## Abstract

**Background:**

Next Generation Sequencing (NGS) is producing enormous corpuses of short DNA reads, affecting emerging fields like metagenomics. Protein similarity search--a key step to achieve annotation of protein-coding genes in these short reads, and identification of their biological functions--faces daunting challenges because of the very sizes of the short read datasets.

**Results:**

We developed a fast protein similarity search tool RAPSearch that utilizes a reduced amino acid alphabet and suffix array to detect seeds of flexible length. For short reads (translated in 6 frames) we tested, RAPSearch achieved ~20-90 times speedup as compared to BLASTX. RAPSearch missed only a small fraction (~1.3-3.2%) of BLASTX similarity hits, but it also discovered additional homologous proteins (~0.3-2.1%) that BLASTX missed. By contrast, BLAT, a tool that is even slightly faster than RAPSearch, had significant loss of sensitivity as compared to RAPSearch and BLAST.

**Conclusions:**

RAPSearch is implemented as open-source software and is accessible at http://omics.informatics.indiana.edu/mg/RAPSearch. It enables faster protein similarity search. The application of RAPSearch in metageomics has also been demonstrated.

## Background

Similarity search is one of the very first computational analyses in the annotation of a genomic/metagenomic dataset. Among many computational tools for this task, BLAST is most commonly used [1,2], owning to its two main advantages: the statistical model for measuring the significance of local sequence similarities [3,4] and its speed. BLAST pioneered the "seed-extension" approach (seed is a match of short subsequences between two sequences, from which the full alignment between the two proteins may be derived by extension), which runs much faster than the rigorous Smith-Waterman local alignment algorithm [5] but achieves approximately the optimal alignment in practice.

The rapid advance of genome sequencing, in particular the NGS techniques that enabled the generation of DNA sequences at several magnitudes higher throughput than the conventional DNA sequencers, has posed new challenges for sequence comparison. For instance, it will take a 1000-CPU computer cluster approximately a month to search a set of DNA sequences of 20G bases total (which can be acquired by one run of a single Illumina/Solexa sequencer) against a protein database of current size (e.g. NCBI NR protein dataset with ~4G amino acids) by using BLAST search. For clarity, we do not specify BLAST programs in this paper--if the input sequences are DNA sequences, BLASTX will be used, which translates DNA sequences in all 6 frames. Note that the current metagenomic approach, i.e. the direct DNA sequencing of environmental species, represents an urgent need for this kind of database searching [6]. In metagenomic studies, BLAST results are used not only for the identification of protein/gene families (thus the functionality of a microbial community) [7,8], but also for the taxonomic analysis of the microbial community (as in MEGAN [9] and Phymm [10]). Without further improvement, database searching will become a bottleneck for the downstream computational analysis of high-throughput sequencing data.

The bioinformatics community was aware of the speed limitation of BLAST tools in the application for the whole genome alignment. Consequently, many ultra-fast tools were developed to replace BLASTN and megaBLAST in aligning long genomic sequences, e.g. Mummer [11], PatternHunter [12,13], BLAT [14] and BLASTZ [15], which typically use sophisticated algorithms to select seeds that can be chained to form long alignments. Most of these novel algorithms, however, are not directly applicable to protein sequence database searching owning to a few distinctions between protein and genomic sequences. Protein sequences (typically of hundreds to thousands residues) are much shorter than genome sequences (typically of millions of bases), while the alphabet of protein sequences (i.e., 20 amino acids) is much larger than that of DNA sequences (i.e., 4 bases). Furthermore, some amino acids (e.g., ILE and LEU) are chemically similar, and often can be replaced by one another without changing the global structure (i.e. the *fold*) or the function of a protein. Hence, the alignment of homologous genomic sequences usually contain relatively long and exact matched seeds (e.g., the maximal exact matches, MEMs [16], or maximum unique matches, MUMs [11], or gapped seeds [12]), whereas the alignment of homologous protein sequences often contain only short exact matches. As a result, in order to achieve high sensitivity, protein database searching tools must retain many false seeds in the fast seed detection step for the seed extension, which is time-consuming.

We proposed a new kind of seeds for protein sequence comparison based on a *reduced (compressed) amino acid alphabet *(wherein similar amino acids are clustered), and developed a new protein database search tool RAPSearch, based on the representation of protein sequences in the reduced amino acid alphabet. Previous studies have shown that the homologous proteins with low sequence identities may still share significant common sequence patterns, for example, the sequence *profiles *[17] (derived from a multiple alignment) or strings of reduced alphabets [18] (derived from individual sequences). RAPSearch follows the seed-extension approach--it first attempts to identify maximal exact matches (MEMs) between the reduced alphabet sequence of a query protein and the reduced alphabet sequence of all proteins in the database using suffix array (see Methods), and then uses the same heuristic extension algorithm as used in BLAST [2] to extend and evaluate each of these seeds.

**Figure 1:**
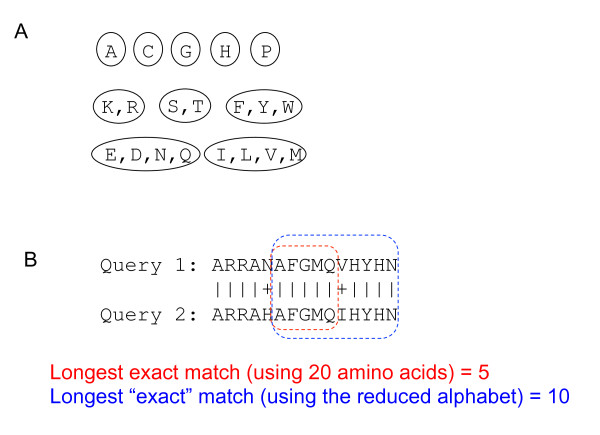
**Speeding up protein database searching by using a reduced alphabet of amino acids**. **(A) **A reduced alphabet with ten symbols, in which, for example, K (Lysine) and R (Arginine) are grouped and represented by a single symbol because of their similar chemical properties. **(B) **The utilization of reduced alphabet will yield longer (and thus more efficient) sequence seeds that are common in homologous proteins. In this example, the maximum exact match (MEM) in the reduced alphabet is of length 10 between the pair of homologous proteins, whereas the MEM in 20-aa alphabet is of length 5. Hence, to retrieve this alignment in the database searching, one must retain the seeds of 10 or longer when using reduced alphabet, and retain the seeds of 5 or longer when using 20-aa alphabet. In this case, the efficiency of the seeds in reduced alphabet is much higher because only the ratio of the number of random seeds in these two cases is about 10^-10^/20^-5 ^≈ 10^-2^.

We illustrate the advantage of using seeds composed of reduced amino acids for protein database searching using a schematic example in Figure 1. In the alignment between a query protein and a subject protein in the database, the longest exact seed match on the 20 amino acid alphabet is 5, whereas the longest exact match on the reduced alphabet shown in Figure 1A is 10. Therefore, in order to identify a protein in the database that is similar to the query protein, we should test all seeds with 5 residues or longer if the 20-aa alphabet is used, whereas only seeds with 10 residues or longer should be retained if the reduced alphabet is used. As a result, approximately 3000 times more seeds on 20-aa alphabet need to be evaluated than the seeds on the reduced alphabet.

We compared the performance of RAPSearch with BLAST, BLAT and HMMER3. BLAT achieves 50 times faster, as compared to BLAST, for protein alignments at sensitivity settings typically used when comparing vertebrate sequences [14]. BLAT was designed to achieve fast similarity search for closely related sequences, and therefore it may not work for detecting remote protein homologs (as shown in our comparison; see Results section). HMMER was developed to achieve accurate detection of remote homologs by using profile Hidden Markov Models (HMM) of protein families as compared to the other database search tools. The new HMMER3 achieves similar speed as BLAST by implementing a new probabilistic model of local sequence alignment and a new heuristic acceleration [19]. Our comparison demonstrates that RAPSearch achieves much faster similarity search than BLAST (and so HMMER3), and its speed is slightly lower than BLAT but achieves much higher sensitivity as compared to BLAT.

## Implementation

RAPSearch adopts the seed-extension approach of BLAST, which identifies the seeds, the maximal exact matches (MEMs) between the reduced alphabet sequence of a query protein and the reduced alphabet sequence of all proteins in the database, followed by extending and evaluating each of these seeds. RAPSearch employs a linear time algorithm to retrieve the MEMs, which first builds a suffix array and a corresponding longest common prefix (LCP) array to index all proteins in the database [20], and then traverses the suffix array based on each query protein. All identified MEMs are subject to a heuristic extension algorithm including an ungapped extension and then gapped extension, similar to BLAST.

### Protein sequence seeds using a reduced amino acid alphabet

The first reduced amino acid alphabet was introduced by Dill in the hydrophobic-polar (HP) model for the study of the folding of globular proteins [21]. Since then, there are more than 50 reduced alphabets of different size that have been proposed for various purposes [22]. A recent study even demonstrated that reduced alphabet is more sensitive and selective in identifying remote homologous proteins [22]. These observations suggested that homologous proteins exhibit a higher sequence identity on the reduced alphabet than that on the 20-aa alphabet, indicating that it is possible to design efficient and sensitive seeds based on a reduced amino acid alphabet.

To select an appropriate reduced amino acid alphabet for RAPSearch, we carried out the following experiments. Using the BaliBase database [23]http://www-bio3d-igbmc.u-strasbg.fr/balibase/, we collected 10,000 pairs of distant homologous proteins that share ~20-40% sequence identify, and 10,000 pairs of proteins from different families (which serve as non-homologous proteins). For each alphabet and each length, we computed the coverage and efficiency of the corresponding seeds: the *coverage *is defined as the fraction of homolog proteins containing at least one seed match, and the *efficiency *is defined as the log ratio of the numbers of homologous and non-homologous proteins containing at least one seed match.

### Seed identification by using suffix array

An essential procedure in RAPSearch (and other seed-extension tools like BLAST, which uses hash table instead of suffix array) is how to choose appropriate seeds for extension (which is more time consuming than finding the seed itself). A commonly used strategy is to define a cutoff for the minimum seed size--a small cutoff may result in a huge amount of seeds to be extended (thus slow down the similarity search), whereas a large cutoff may miss some seeds that otherwise may lead to significant alignments. BLAST uses minimum size of 3 (residues for proteins) but also requires that there are two seeds in the same diagonal that span no more than a certain length. RAPSearch will extend all single seeds that have been identified by looking up in the suffix array of target protein sequences. As RAPSearch uses reduced alphabets to present proteins, RAPSearch can use longer seed cutoff, thus achieving faster similarity search.

### Minimal seed selection algorithm

RAPSearch generates seeds of a minimal length of 6-9 amino acids, with longer seeds for frequent words and shorter seeds for rare words. RAPSearch decides the minimum length of seeds starting at a particular query position based on the frequency of the 6-mers starting at that position. Once the minimum length of the seeds is selected for a particular position, all the seeds of at least the required length will be retrieved by looking up in the pre-computed suffix array of protein similarity search database. The minimum seed length selection algorithm is shown as follows (for a given position *i *in a query sequence).

Seed length selection algorithm (position i):

minseed ← 6

addlen ← 0

hexmerF ← the frequency of the 6-mer starting at position i

medianF ← the median of the frequencies of all 6-mers in the protein database

expectF ← hexmerF

if (expectF > medianF):

# aaComp(k) is the frequency of the corresponding amino acid at position k

while expectF * aaComp(i + addlen + 1) < medianF:

addlen ← addlen + 1

expectF ← expectF * aaComp(i + addlen + 1)

minseed ← minseed + addlen

return minseed

### Seeds with mismatches

We further consider seeds with mismatches (these mismatches that can not be handled by reduced alphabets). Long seeds (at least 10 aa) that allow at most one mismatch either at position 3, 4, 5, or 6 as in the following patterns, OOOXOOOOOO, OOOOOOXOOO, OOOOXOOOOO and OOOOOXOOOO (where X indicates a mismatch, and Os indicate exact matches). We replace the residue at each of the positions allowing mismatches (marked with X) by one of the reduced amino acids in the reduced alphabet in turn to search for exact matches, achieving identification of seeds with mismatches using suffix array.

### Ungapped and gapped alignment

We implemented ungapped and gapped extension procedures following the same approach used in BLAST [2].

### Statistical significance evaluation

We used the statistical evaluation method from BLAST, and used the same model and parameters (for BLOSUM62 substitution matrix) as BLAST to evaluate the significance of the resulting local alignment.

### Protein similarity search databases and other datasets

We tested RAPSearch on several public metagenomic datasets with various read lengths [7,24]. The nucleotide sequences were downloaded from the NCBI short read archive, and the MG-RAST server http://metagenomics.nmpdr.org/. The protein similarity search databases we used include a 98% non-redundant dataset (prepared by using CD-HIT [25]) of protein sequences from prokaryotic genomes, plasmid and viral genomes, collected in the IMG 3.0 http://img.jgi.doe.gov, eggNOG database (of sequences that have COG annotations) (downloaded from http://eggnog.embl.de/), and NCBI non-redundant (nr) database (downloaded from NCBI ftp site). The complete genomes (*Escherichia coli *K12 substr MG1655, NC_000913; *Salmonella typhi*, NC_003198; and *Desulfococcus oleovorans *Hxd3, NC_009943) and their gene annotations we used for the simulation study were downloaded from the NCBI ftp site.

### Other computational tools

RAPSearch was compared to BLAST, BLAT and HMMER. The BLAT source codes were downloaded from http://hgwdev.cse.ucsc.edu/~kent/src/blatSrc34.zip. The default filtering option in BLAST automatically masks low complexity regions of amino acids by using the SEG approach [26] prior to similarity search. The SEG masking is also implemented in RAPSearch. For comparison purpose, SEG was also applied to the six frame translations of the short reads for (protein) BLAT similarity search.

## Results

### Selection of reduced amino acid alphabet

**Figure 2:**
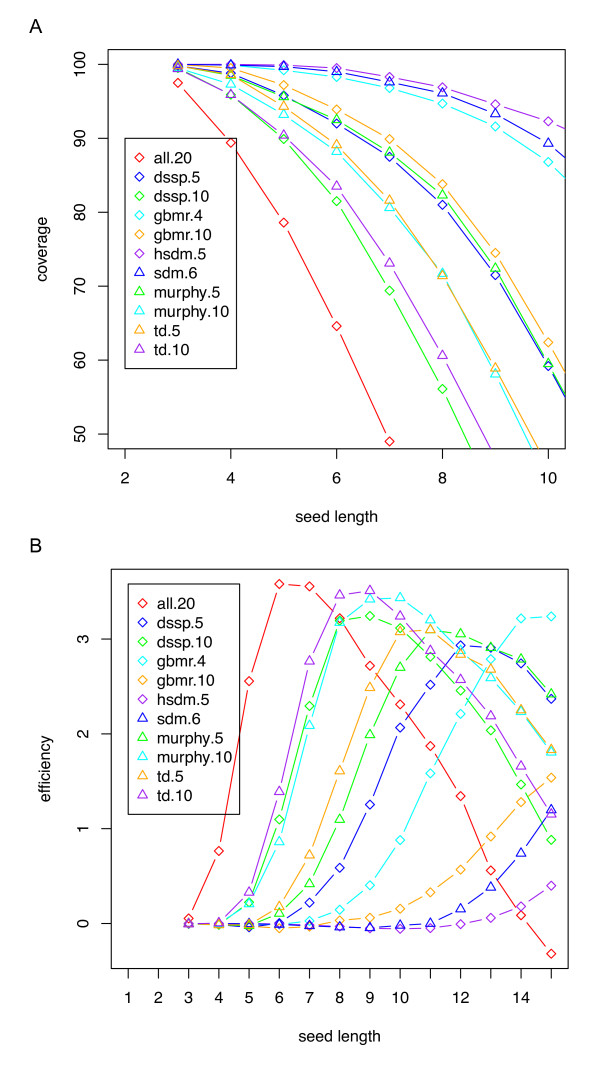
**Comparison of the performance of different reduced amino acid alphabets**. The performance of a reduced alphabet of amino acids is measured by the coverage of seeds in the alphabet, defined as the percentage of pairs of alignments (of distantly related proteins of 20%-40% identify) that have at least one of the seeds of certain length **(A)**, and the efficiency, defined as log ratio of the percentage of pairs of alignments that have at least one of the seeds of a defined length and the percentage of any pairs of unrelated sequences that share at least one of the seeds **(B)**. See Table 1 for details of the alphabets. Note that only a subset of the alphabets is shown in the figure for clarity.

We started with the testing of the performance of different reduced amino acid alphabets to select an appropriate reduced amino acid alphabet for seed detection. Desirable seeds are those that can be found in homologous proteins, but not in unrelated proteins. We tested more than 50 reduced amino acid alphabets that have been proposed for various purposes (from the studies of protein folding [21], to protein design [27,28], and sensitive fold recognition [22]) (see Table 1 for a list of reduced alphabets mentioned in this paper). For each alphabet, we calculated its coverage and efficiency at seed length ranging from 3 to 15. As shown in Figure 2, seeds in highly compressed alphabets (e.g., gbmr.4, which has 4 letters representing 4 groups of amino acids) generally have higher coverage than seeds in alphabets of larger sizes (e.g., gbmr.10 with 10 groups of amino acids), although the coverage varies for alphabets of the same size that are derived using different methods (e.g., hsdm.5 has higher coverage than dssp.5) (Figure 2A). This is consistent with previous observations that highly compressed alphabets such as gbmr.4 achieved higher sensitivity in fold recognition [22]. But highly compressed alphabets also tend to show low efficiency (i.e., they result in seeds that can often be found between two non-homologous sequences) (Figure 2B) so that they are not appropriate for fast database searching. Based on these results, a reduced amino acid alphabet (called murphy.10, which has 10 letters and was derived based on the BLOSUM matrix; see Table 1) [29] was picked as the reduced alphabet for seed identification by RAPSearch, aiming to achieve the greatest speedup while keeping minimal loss of sensitivity.

**Table 1:** A list of reduced amino acid alphabets

Alphabet	Size of the alphabet	Amino acid groups
all.20	20	P G E K R Q D S N T H C I V W Y F A L M
dssp.5	5	[AEHKQR] [FILMVWY] [CST] [DN] [GP]
dssp.10	10	[EKQR] [IV] [LY] F [AM] W [HT] C [DNS] [GP]
gbmr.4	4	G [ADEKNQRST] [CFHILMVWY] P
gbmr.10	10	G D N [AEFIKLMQRVW] Y H C T S P
hsdm.5	5	[LIVFMY] W C [DNTSKEQRAGP] H
sdm.6	6	[YFLIVM] C W [DNTSQKERAG] H P
murphy.5	5	[LVIMC] [ASGTP] [FYW] [EDNQ] [KRH]
murphy.10	10	A [KR] [EDNQ] C G H [ILVM] [FYW] P [ST]
td.5	5	[PG] [EKRQ] [DSNTHC] [IVWYF] [ALM]
td.10	10	P G [EKRQ] [DSN] T [HC] [IV] [WYF] A [LM]

The alphabets were downloaded from http://www.rpgroup.caltech.edu/publications/supplements/alphabets/HP/Welcome.html.

### Selection of seed lengths

We also observed that, given a protein sequence database, words of the same length (e.g., hexamers) are of very different frequencies in the database because of the composition bias (some amino acids are more frequent than others), with a small number of extremely frequent words. For example, in a protein database that has 1,231,398,422 amino acids (collected from IMG 3 database; see Methods), the most frequent hexamer has 1,351,564 copies, whereas the median frequency of all hexamers is a mere 176 (see Supplementary Figure 1 in Additional File 1 for the frequency distribution for the hexmers). If a fixed minimal seed length (e.g., 6) was used, the matches between a few extremely frequent words would dominate the seed pool, the majority of which will not lead to meaningful, long alignments. Instead, we used the minimal seed selection algorithm to automatically determine the minimum length of the seeds starting at a particular position of a query sequence based on the frequency of its first 6 residues--which can be retrieved from a pre-computed lookup table quickly--and the amino acid composition for the positions after. Using this strategy, seed matches of length 6-9 will be detected by RAPSearch among query sequences and the protein sequences in the database. This length range 6-9 works best in practice, and Figure 2B also shows this is where the seeds (of murphy.10 reduced alphabet) achieve highest efficiency.

### Comparison of RAPSearch with BLAST and BLAT

We tested RAPSearch on several published metagenomic datasets [7,24] with various read lengths to demonstrate its performance for short reads acquired by different NGS-sequencers. RAPSearch achieved ~20-90 times speedup as compared to BLAST, with a small loss of sensitivity (Table 2). The results suggest that RAPSearch gained its speedup by more efficiently selecting seeds from pairs of homologous proteins. The speedup is more significant for shorter reads. For example, RAPSearch is > 90 times faster than BLAST for the SRR020796 dataset (average read length = 72 bp). By contrast, RAPSearch only achieved ~20 times speedup for the TS28 dataset, which has reads of ~320 bp and >70% of the reads have similarity hits in the IMG database. This contrast can be partially explained by: 1) a relatively smaller proportion of reads will have homologs for shorter reads (~19% of the SRR020976 reads have homologs detected) [30]; and 2) shorter reads require significantly less gapped extension as compared to longer reads, and gapped extension is more time consuming than ungapped extension. Here RAPSearch was compared to BLAST programs in blast2.1.18. Note that BLAST in a newer blast package (blast+-2.2.23, a version rewritten in C++), denoted as BLAST+, did not produce significantly different results on the datasets we tested, but the running time is more than doubled (e.g., the running time of BLAST+ is ~330 CPU hours as compared to ~150 CPU hours for BLAST on the 4440037 dataset).

**Table 2:** The performance of RAPSearch as compared to BLAST.

Test datasets	Number of reads	Read length (nt)	Reads with homologs (by BLAST)	Running time (CPU hours)		**Reads with homologs found in the IMG protein database**^**a**^		
				
				BLAST	RAPSearch	**Overlap**^**g**^	BLAST-only	RAPSearch-only
SRR020796 (2%) ^b^	1,164,805	72	19%^e^	1,590 ^f^	16.8	218,134 (98.4%)	2,832 (1.3%)	745 (0.3%)
4440037^c^	188,445	100	5%	154	3.5	9,791 (95.3%)	270 (2.6%)	213 (2.1%)
TS50^d^	622,554	200	75%	1000	54.3	459,509 (97.9%)	7,339 (1.5%)	2683 (0.6%)
TS28^d^	312,665	329	75%	900	45.7	225,953 (96%)	7,511 (3.2%)	1,222 (0.5%)

^a^: the reads are searched against the 98% non-redundant dataset of proteins collected in the IMG database with a total of 4,054,694 proteins, and an E-value cutoff of 1e-1 was used to define homologs (less stringent) for the Illumina reads (the SRR020796 dataset) considering the reads are extremely short, and an E-value cutoff of 1e-3 for the rest. ^b^: the dataset was downloaded from the NCBI website (from the rumen microbiota response study), and only 2% of the reads were used for testing because the BLAST search of the entire dataset will require a computer farm. ^c^: dataset was from the nine biomes project [7]. ^d^: TS50 (4440615.3) and TS28 (4440613.3) datasets were from the Twin Study [24]. 4440037, TS50 and TS28 datasets were downloaded from the MG-RAST server. ^e^: the percentage of reads that have homologs in the IMG database as identified by BLAST. ^f^: the running time was estimated based on the running time of BLAST search of a small fraction of the original dataset on the same computer (Intel Xeon 2.93 GHz) on which RAPSearch was carried out for comparison purposes; the actual BLAST search of the original datasets was carried out on BigRed, a computer cluster maintained at Indiana University. ^g^: the Overlap column lists the total number of reads that have homologs in the IMG database detected by both BLAST and RAPSearch, while the total number of reads that have homologs in the IMG database detected by BLAST or RAPSearch only are listed in the BLAST-only and RAPSearch-only column, respectively.

**Figure 3:**
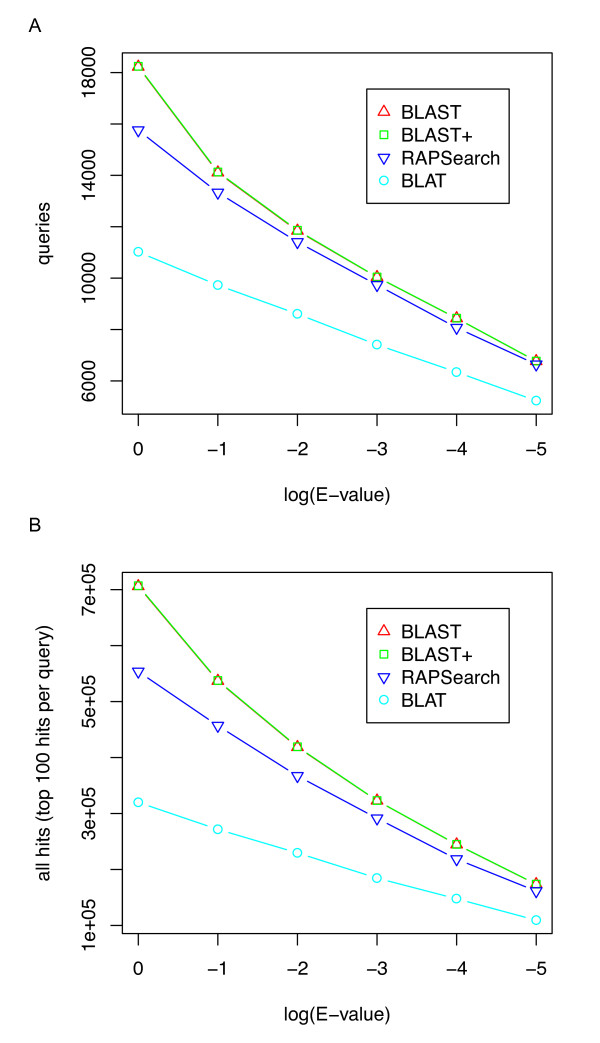
**Comparison of the similarity search sensitivity at different E-value cutoffs**. RAPSearch, BLAST (and BLAST+, using default parameters), and (protein) BLAT were compared on the same query dataset (4440037). The total number of queries that have at least one homolog in the IMG protein sequence database (based on the corresponding E-value cutoff) was used in **(A)**, whereas the total number of all significant hits (up to 100 hits per query) was used in **(B)**. Note that BLAST and BLAST+ have almost identical sensitivity (but BLAST+ is twice as slow). For this dataset search, BLAST, RAPSearch and BLAT used 154, 3.5 and 2.7 CPU hours (on Intel Xeon 2.93 GHz), respectively.

The detailed comparison of the performances by BLAST (and BLAST+), BLAT and RAPSearch on one query dataset is shown in Figure 3. (See Supplementary Figures 2 and 3 in Additional File 1 for detailed comparison for the TS28 and TS50 datasets.) RAPSearch tends to miss some distant similarities, but better captures closely related proteins. Under the stringent E-value cutoffs (e.g, 1e-3 or 1e-5 as used in most metagenomic studies [7]), RAPSearch has minimal loss of sensitivity as compared to BLAST. By contrast, BLAT tends to miss more similarity hits (Figure 3). Note that the difference at the query level (e.g., how many queries have significant hits as seen in Figure 3A) is smaller than the difference at the level of individual hits (Figure 3B). We also tested the performance of RAPSearch as compared to BLAST when searching against different protein databases, and the results showed consistent speedup by RAPSearch (see Table 3). We examined some of the similarities that are missed by RAPSearch--they are usually due to the lack of proper seeds between the query and the subject protein sequences. Interestingly, RAPSearch also detected some homologous proteins that are missed by BLAST search. And there is no obvious significance (measured by E-value) difference between the unique hits detected by either RAPSearch or BLAST (but not both) (see Supplementary Figure 4 in Additional File 1). An example of similarity detected only by RAPSearch is shown in Supplementary Figure 5 (Additional File 1).

**Table 3:** Performance comparison of similarity search tools on the same query dataset (4440037) against different protein similarity search databases.

Database	Total sequences	Total aa	Running time (CPU hours)		Reads with homologs found in the protein database (E-value cutoff = 1e-3)		
			
			BLAST	RAPSearch	Overlap	BLAST-only	RAPSearch-only
Extended COG ^a^	670,804	215,687,522	27.5	0.6	6384 (94.4%)	259 (3.8%)	123 (1.8%)
IMG	4,054,690	1,231,432,735	154	3.5	9,791 (95.3%)	270 (2.6%)	213 (2.1%)
NR ^b^	8,994,603	3,078,807,967	428	9.7	10546 (95.5%)	256 (2.3%)	238 (2.2%)

^a^: Extended COG contains sequences collected in eggNOG database; ^b^: NR is the NCBI non-redundant database. The total number of sequences and amino acids included in each database are shown in the "Total sequences" and "Total aa" columns, respectively.

### Comparison of RAPSearch with HMMER3

We were only able to compare RAPSearch with HMMER3 on one similarity search database, the extended COG database (eggNOG database) [31], for which we built HMMs of the COG families using Muscle [32], a multiple alignment program, and the HMM builder from the HMMER3 package. (A direct comparison of RAPSearch with HMMER3 is difficult for other similarity search databases, as HMMER is based on profile HMMs of protein families whereas RAPSearch searches against a protein sequence dataset.) The comparison shows that HMMER3 does not necessarily achieve higher sensitivity as compared to RAPSearch (and BLAST) for the short reads at the same similarity significance level. For example, for the query dataset 4440037, in total 5,975 reads have annotations at an E-value cutoff of 1e-3 based on the HMMER3 search results, whereas in total 6,230 reads have annotations based on the RAPSearch search results. In addition, HMMER3 may be too slow (although it is as fast as BLAST) for some applications, such as the similarity search of large datasets of short reads.

### Evolutionary distance matters

Detecting distant homologs is difficult for sequence-based comparison methods; and detecting distant homologs using short reads is even more challenging [30]. We simulated short reads from complete genomes and then applied similarity searches to show the impact of sequence divergence on the performance of similarity search tools. As shown in Table 4, none of the methods we tested could detect all homologs based on similarity search of simulated short reads. Overall, BLAST shows higher sensitivity than RAPSearch and BLAT on detecting distant homologs, i.e., similar proteins from evolutionarily distant species (e.g., the same phylum but different subphylums), whereas RAPSearch achieved comparable sensitivity as BLAST on detecting close homologs, i.e., homologous proteins from evolutionarily close species (e.g., same family but different genera). And RAPSearch achieved higher sensitivity than BLAT for detecting both close and distant homologs using short reads. We expect that RAPSearch will become more useful for annotating metagneomic datasets as more microbial genomes are being sequenced, resulted from, for example, the effort of sequencing reference genomes from human microbiome [33].

**Table 4:** Comparison of the factions of reads with homologs at different evolutionary distances that are detected by different similarity search tools.

Evolutionary distance	**Apply E-value cutoff?**^**c**^	BLAST	RAPSearch	BLAT
family^a^	No	0.95	0.88	0.77
	E-value = 0.1	0.91	0.87	0.76
	E-value = 0.001	0.86	0.84	0.71

phylum^b^	No	0.79	0.51	0.21
	E-value = 0.1	0.68	0.48	0.16
	E-value = 0.001	0.48	0.39	0.09

^a^: short reads of ~100 bps simulated from the gene sequences of *Salmonella typhi *were searched against the proteins of *Escherichia coli *K12 (*Salmonella typhi *and *Escherichia coli *belong to the same family, but different genera). ^b^: short reads of ~100 bps simulated from the gene sequences of *Desulfococcus oleovorans *Hxd3 were searched against the proteins of *Escherichia coli *K12 (*Desulfococcus oleovorans *and *Escherichia coli *belong to the same phylum, but different subphylums). Only the genes of at least 90 bp (encoding 30aa) were included in the statistics, and two genes with 40% or higher amino acid identity spanning at least 50% of the length of one gene were considered as *homologs*. ^c^: "no" indicates that no E-value cutoff was applied to filter out the similarity hits for the short reads, and E-value = 0.1 indicates that only similarity hits with E-value < = 0.1 were included for the statistics.

## Conclusions

The comparison of RAPSearch with other tools demonstrates that RAPSearch can be used to achieve a fast protein similarity search with minimal loss of sensitivity. This improvement relieves the computational need for database search for short reads that are derived from NGS techniques. Instead of running BLAST searches on a computer farm with many CPUs, a RAPSearch search against a large protein database (such as the IMG database) for a dataset from a single run of 454 sequencer or Illumina sequencer can be achieved on a single PC with multiple cores, or a small computer cluster.

The tests of RAPSearch on detecting similarities at different evolutionary distances (results summarized in Table 4) showed that RAPSearch and BLAT missed more similarity hits between distantly related proteins. The same trend was observed in the tests on real metagenomic datasets (Figure 3), in which RAPSearch missed proportionally more hits at more stringent E-value cutoffs (the sensitivity loss is even worse for BLAT). This problem may be alleviated when more reference genomes become available and more proteins are added to the similarity search database.

A simple calculation shows that RAPSearch can potentially achieve >120 (10^6^/20^3^) speedup as compared to BLAST: a seed (of a minimal size of 3) can be found in 1 out of 20^3 ^possible matches in BLAST, and a seed can be found in 1 out of 10^6 ^matches in RAPSearch (which uses seeds of 6 amino acids or longer in the murphy.10 alphabet of size 10). The actual speedup varies, depending on the read length and the nature of the microbial community from which a metagenomic dataset is derived. Generally, RAPSearch achieved more significant speedup on shorter reads, and on the datasets derived from communities with better-characterized microbial organisms (such as the human-associated microbial communities, as in the TS50 and TS28 datasets). RAPSearch achieved ~20-90 times speedup as compared to BLAST on the metagenomic datasets that we tested; this represents the speedup that RAPSearch can achieve on a typical metagenomic dataset in practice.

We will work on several improvements of RAPSearch, aiming to further accelerate the similarity search. The first improvement is to implement a new version of RAPSearch that supports multiple threads, best utilizing the multiple cores that a modern computer typically has. The second strategy we will try is to pre-process the queries to eliminate redundant similarity searches.

## Availability and Requirements

RAPSearch (which stands for *R*educed *A*lphabet based *P*rotein similarity *Search*) was implemented in C++, and has been tested extensively in linux platforms. The inputs of RAPSearch can be either amino acid sequences or nucleotide sequences (which will be translated in 6 frames). RAPSearch produces result files that are similar to BLAST outputs. RAPSearch source codes are available as Supplementary Software (Additional File 2). The details of the comparison between the BLAST and RAPSearch search results, and the source codes of RAPSearch can be found at the RAPSearch website, http://omics.informatics.indiana.edu/mg/RAPSearch.

## Authors' contributions

Ye and Tang designed the algorithm. All authors were involved in the implementation of the algorithm and writing the manuscript. All authors read and approved the final manuscript.

## Supplementary Material

Additional file 1 **Supplementary table and figures**. The file contains Supplementary Table 1, and Supplementary Figures 1-5. Click here for file

Additional file 2 **RAPSearch package**. A package of RAPSearch source codes, implemented in C++. Click here for file
