# Supplementary material for: RAPSearch: a fast protein similarity search tool for short reads
Source: BMC Bioinformatics. 2011 May 15;12:159. doi: 10.1186/1471-2105-12-159 (PMC3113943; doi:10.1186/1471-2105-12-159)
Supplement: Additional file 1 — Supplementary table and figures. The file contains Supplementary Table 1, and Supplementary Figures 1-5. [file 1471-2105-12-159-S1.PDF]

RAPSearch: a Fast Protein Similarity Search Tool for Short Reads

Yuzhen Ye, Jeong-Hyeon Choi, Haixu Tang

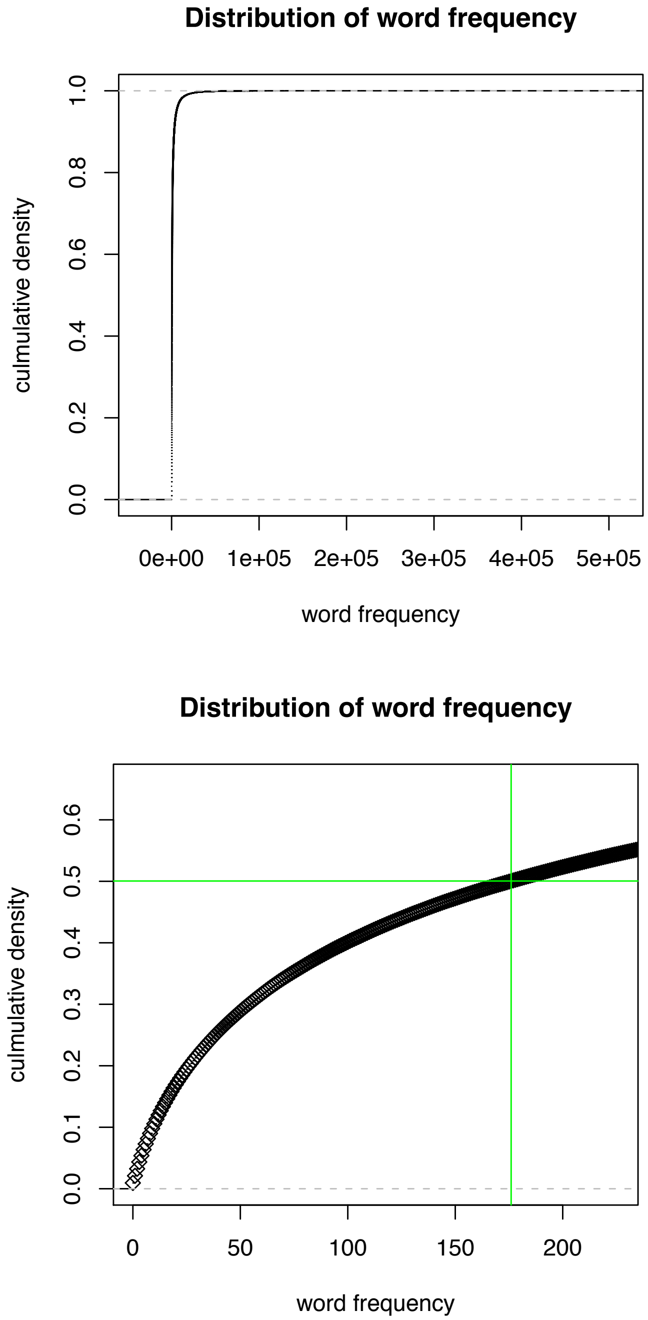

Supplementary Figure 1. The distribution of word frequency: (a) overall, (b) zoom in at the low frequency region. The vertical green line indicates the median frequency.

(a)

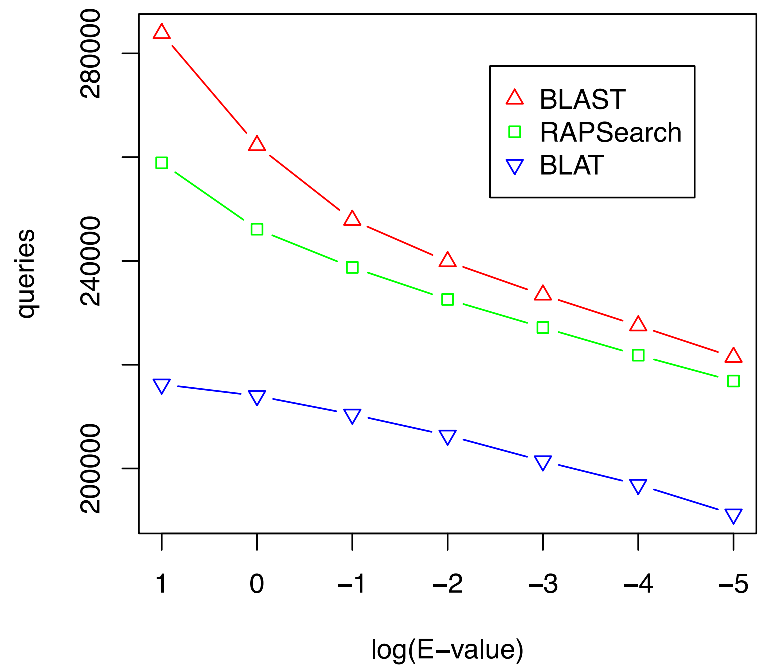

(b)

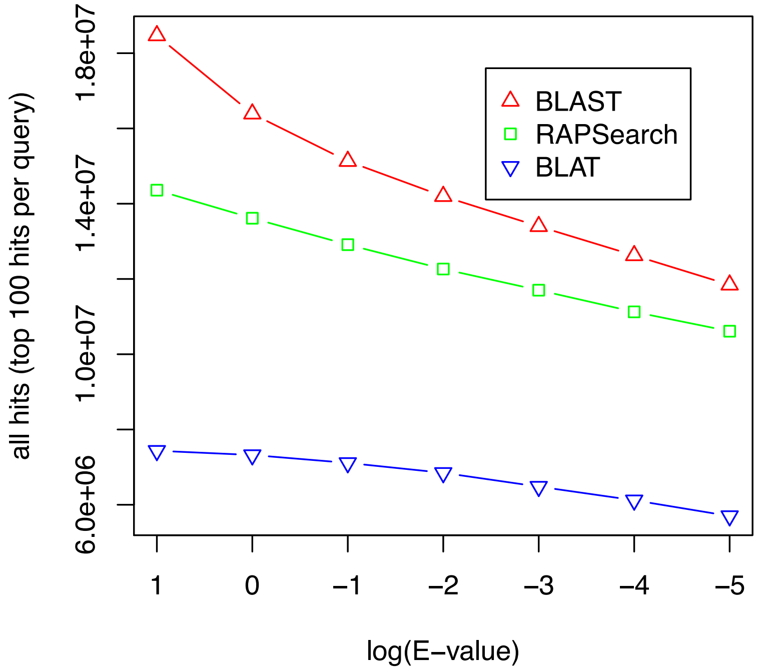

Supplementary Figure 2. Comparison of the performance of RAPSearch, BLAST, and BLAT on the query dataset TS28 at different E-value cutoffs. The total number of queries that have at least one homolog in the IMG protein sequence database (based on the corresponding E-value cutoff) was used in (a), whereas the total number of all significant hits (up to 100 hits per query) was used in (b).

(a)

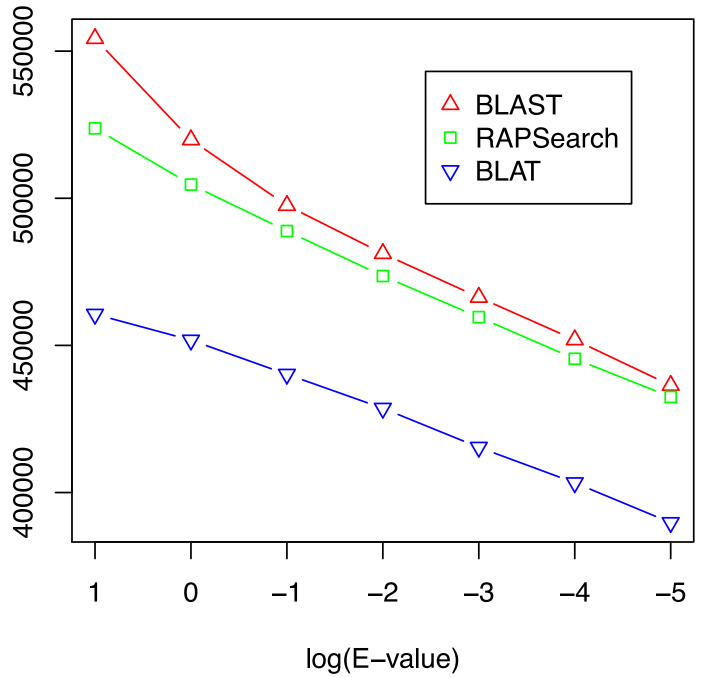

(b)

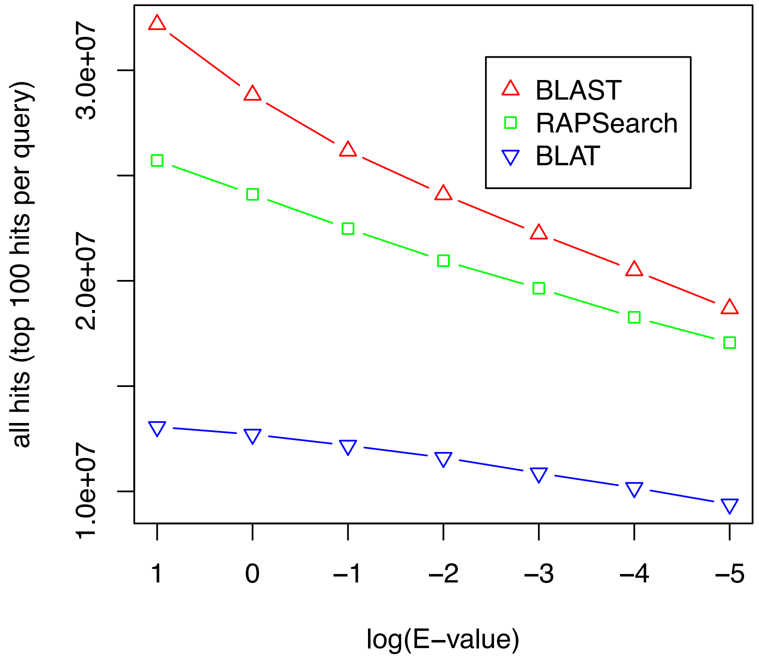

Supplementary Figure 3. Comparison of the performance of RAPSearch, BLAST, and BLAT on the query dataset TS50 at different E-value cutoffs. The total number of queries that have at least one homolog in the IMG protein sequence database (based on the corresponding E-value cutoff) was used in (a), whereas the total number of all significant hits (up to 100 hits per query) was used in (b).

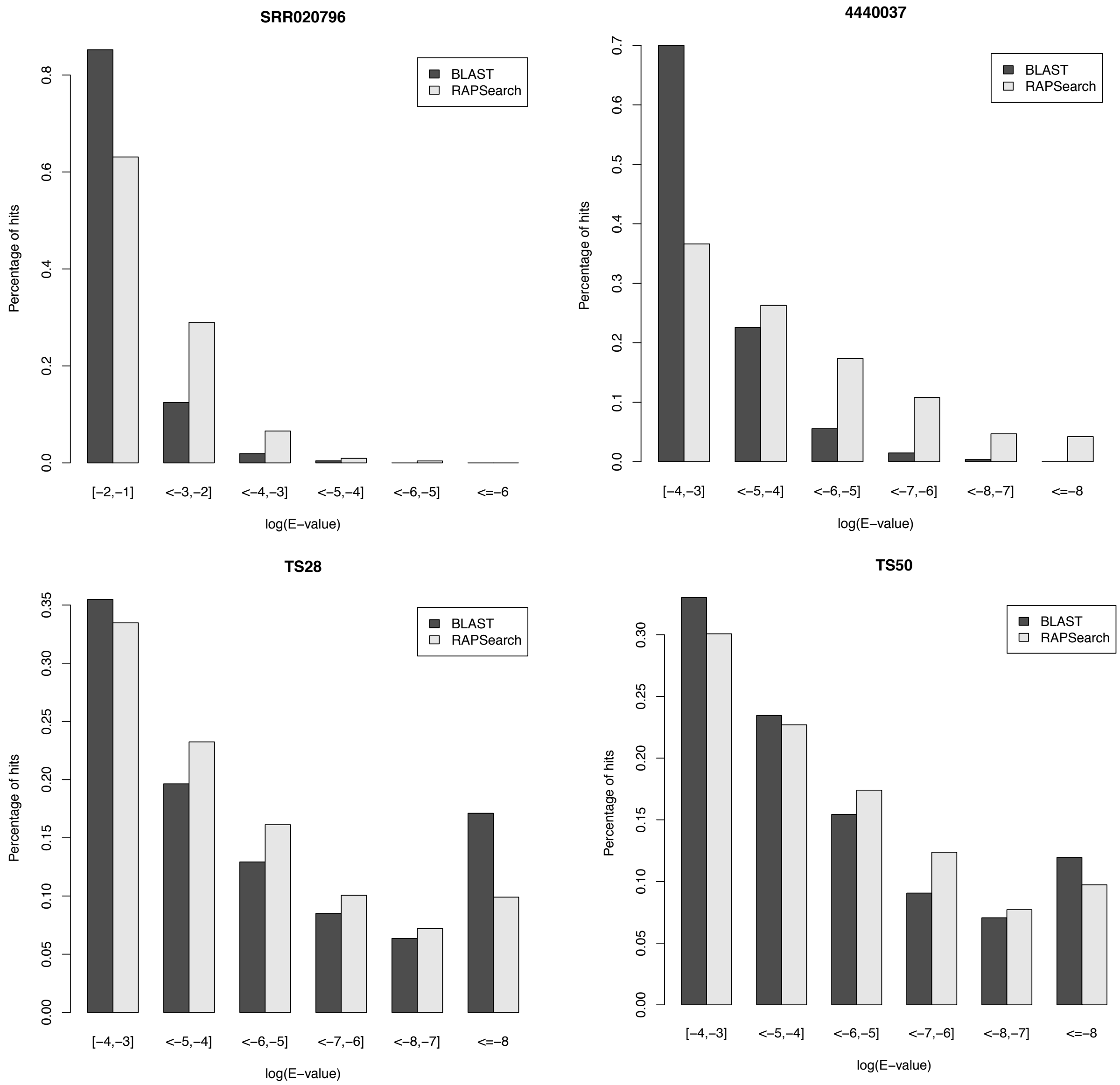

Supplementary Figure 4. The E-value distributions of similarity hits that were detected by either BLAST or RAPSearch but not both.

```
>2986919 259536.Psyc_0853 Evalue=-3.56 Identity=76.00
Query: 78   LGATLAGMAFINASVGAIHALSYPL 4
Sbjct: 225  LGSTLAGIAFVNASVAAVHGLSYPL 249
```

Supplementary Figure 5. An example of sequence similarity detected only by RAPSearch (but not BLAST). In this case, the longest seed found by RAPSearch has length of 12 residues (highlighted in bold) with two mismatches (M versus I, and I versus V; but I, M, and V are reduced to the same letter when using the reduced amino acid alphabet).
